# Comparison of half-dose alteplase and LMWH in intermediate-high risk pulmonary embolism: a single-center observational study

**DOI:** 10.1186/s43044-025-00669-5

**Published:** 2025-07-25

**Authors:** Ömer Selim Selim Unat, Pervin Korkmaz, Akın Çinkooğlu, Özge Can, Elton Soydan, Selen Bayraktaroğlu, Gürsel Çok, Recep Savaş, Funda Karbek Akarca, Sanem Nalbantgil, Celal Çinar, Mehmet Uyar, Kubilay Demirağ, Tahir Yağdi, Çağatay Engin, Münevver Erdinç, Feza Bacakoğlu

**Affiliations:** 1 Dr. Suat Seren Göğüs Hastalıkları Hastanesi, Izmir, Turkey; 2 Ege University, Izmir, Turkey; 3 Istanbul Medicana International Hospital, Istanbul, Turkey

**Keywords:** Pulmonary embolism, Intermediate-high risk, Thrombolysis, Half-dose alteplase, Low molecular Weight heparin, Real-life study

## Abstract

**Background and aim:**

The use of thrombolytics in intermediate-high risk pulmonary embolism (PE) remains controversial. This study evaluated the efficacy and safety of half-dose alteplase compared to anticoagulation with LMWH in this group.

**Material and methods:**

Patients treated with thrombolytics (50 mg alteplase) after the establishment of EGEPET (2.10.2018) formed the prospective group, while the retrospective group included patients treated with LMWH (enoxaparin) before EGEPET. Primary outcomes were one-month and one-year mortality. Secondary outcomes were vital sign changes after thrombolysis, hemorrhagic events, recurrence of embolism, chronic pulmonary thromboembolism (CPTE), and chronic thromboembolic pulmonary hypertension (CTEPH).

**Results:**

Thrombolytic group (n = 59) and anticoagulation group (n = 38) were similar in age, comorbidities, and vital signs, except for higher pulse rates in the thrombolytic group. In the thrombolytic group, PaO₂/FiO₂ ratio significantly improved [330 (270–380) to 417 (351–447), *p* < 0.001], and pulse rate decreased [116 (105–127) to 91 (80–104), *p* < 0.001]. In the anticoagulation group, oxygenation showed no significant change, but pulse rate improved. No major bleeding occurred in either group. One-month mortality was 6.7% in the thrombolytic group and 15.8% in the anticoagulation group (*p* = 0.18). One-year mortality was 13.7% and 26.3%, respectively (*p* = 0.17). Advanced age (> 67) (OR: 8.82, %95 CI 1.54 – 50.53 *p* = 0.014) and elevated second-day pulse > 94/min (OR 7.61, 95% CI 1.33–43.49, *p* = 0.022) were independent predictors of one-month mortality in the multivariate analysis.

**Conclusion:**

Thrombolytic therapy significantly improved oxygenation and clinical findings without major complications. Although mortality rates were lower in the thrombolytic group, the difference was not statistically significant. These results should be interpreted with caution, and larger prospective studies are needed to confirm the clinical efficacy and safety of thrombolytic therapy in this patient population.

## Introduction

Acute pulmonary embolism (PE) is a disease with high mortality and requires rapid intervention. The increased pulmonary vascular resistance due to filling of pulmonary arteries with embolism, right heart ventricle failure and hemodynamic decompensation can cause death [1].

According to European guidelines, acute PE is classified by mortality risk into low, intermediate, and high categories. Intermediate risk is further divided into intermediate-low and intermediate-high. Intermediate-high risk PE is defined as the presence of right ventricular (RV) dysfunction (detected by echocardiography (ECHO) or CTPA) along with positive cardiac biomarkers (e.g., elevated troponin), in the absence of systemic hypotension or shock [2]. Although intermediate-high risk PE is less common (9.6%), it is associated with high mortality. Therefore, guidelines recommend evaluating these hemodynamically stable patients with right ventricular failure as a distinct group [2, 3].

The study comparing tenecteplase and unfractionated heparin in patients with radiological evidence of right ventricle failure (computed tomography pulmonary angiography or ECHO) and having cardiac injury documented by troponin elevation, has found that hemodynamic decompensation occurs less in the thrombolytic group, but bleeding is detected at higher rates [4]. In the meta-analysis which contains patients with intermediate risk, thrombolysis has not provided additional benefits also it increases bleeding risk compared to anticoagulation treatment [5]. Another study has shown that there is no statistically significant difference between the efficacy of thrombolysis and low molecular weight heparin (LMWH) in hemodynamically stable patients with RV failure without the evaluation of cardiac biomarkers [6]. According to these results, thrombolytic therapy in this population may reduce the incidence of hemodynamic collapse and improve right ventricular function, which are important clinical endpoints. However, these potential benefits should be weighed against the increased risk of major bleeding and intracranial hemorrhage [4, 5].

In these studies, risk classification was not made fully in accordance with the last PE guideline. There is no sufficient and accurate data about the treatment of intermediate-high risk PE patients in the literature. Hence, this study aimed to compare the administration of “half-dose alteplase” and LMWH in intermediate-high risk PE patients by evaluating the effect of half-dose alteplase on one-month and one-year all-cause mortality, changes in post-thrombolysis vital signs, and treatment complications.

## Materials and methods

### Patient population

This observational, single-centered study was designed with prospective and retrospective stages. The patients diagnosed with PE at Medical Faculty Hospital and classified as intermediate-high risk group according to the 2019 ERS/ESC guidelines were included in the study. Ege Pulmonary Embolism Team (EGEPET) was established on 2.10.2018.

Prospective thrombolytic group was composed of the patients evaluated at EGEPET between 02.10.2018–13.02.2023 and treated with half-dose thrombolysis. Prior to the establishment of EGEPET, patients who were diagnosed with intermediate-high PE between 01.1.2016–2.10.2018 and were treated with only LMWH were included in the study retrospectively (anticoagulation group or historical control group).

The EGEPET, which was founded as Pulmonary Embolism Rescue Team at “Ege” Medical Faculty, is a multidisciplinary team composed of pulmonologists, cardiologists, emergency medicine specialists, cardiovascular surgeons, intensive care physicians, and radiologists. The team operates on a 24/7 basis and maintains continuous communication through a secure mobile-based instant messaging platform that enables real-time consultation and rapid decision-making. This system facilitates early diagnosis, expedited risk stratification, and the collaborative management of acute PE cases, including consensus on advanced interventions such as systemic thrombolysis or catheter-directed thrombolysis.

*Inclusion Criteria:* Patients aged 18 years or older, whose PE was confirmed with computed thorax pulmonary angiography (CTPA) or ventilation/perfusion (V/P) scintigraphy, patients classified as intermediate-high risk in terms of early mortality.

*Exclusion Criteria:* Patients with missing data, undocumented right ventricle failure, contraindications for thrombolysis, full-dose thrombolysis due to hemodynamic instability, dose escalation from half to full thrombolysis during follow-up, high bleeding risk as determined by EGEPET, or unstable comorbidities affecting mortality (e.g., malignancy, poor general condition) were excluded.

After diagnosing PE, the risk classification was made. The intermediate-high risk group was defined as patients with sPESI (simplified Pulmonary Embolism Severity Index) score ≥ 1 and evidence of right ventricular failure on ECHO or CTPA and elevated troponin or patients with sPESI score 0 and right ventricular dysfunction detected by imaging methods and biomarker. The risk classification of the patients was discussed and decided at EGEPET. The early mortality risk for the patients before the establishment of EGEPET was performed retrospectively according to ESC/ERS PE guideline [2]. RIETE and HAS-BLED scores were calculated and recorded to assess bleeding risk. [7, 8].

### Ethical considerations

All participants provided written informed consent prior to inclusion in the study. The study was approved by the Clinical Research Ethics Committee of the university’s Faculty of Medicine (22–4.3/26) and by the Ministry of Health, Medicines, and Medical Devices Agency (21-AKD-107). Consent was obtained from the patients to participate in the study. Our study is an observational drug study. A clinical trial registration number has been obtained: NCT05512702.

### Treatment decision and method of application

Patients in the thrombolysis group were not randomized; the decision to administer thrombolytic therapy was made by the EGEPET based on clinical judgment and the individual patient’s medical condition. As thrombolytic therapy for prospective group, 50 mg alteplase was administered by intravenous infusion over two hours. After alteplase, the anticoagulant treatments of patients were continued by enoxaparin.

In historical control, providing that kidney function test was not impaired, anticoagulation was used as 1 mg/kg 12 hourly enoxaparin was administered subcutaneously.

### Evaluation of complications

Hemorrhagic stroke, major bleeding, minor bleeding, and heparin-associated thrombocytopenia (HIT) were evaluated as treatment complications.

Major bleeding was defined as hemorrhage in critical organs (e.g., intracranial, intraocular, pericardial), or a ≥ 2 g/dL drop in hemoglobin accompanied by clinical consequences such as the need for transfusion, prolonged hospitalization, or death within 7 days. A hemoglobin drop alone without clinical significance was not considered sufficient to classify an event as major bleeding. Minor bleeding was also assessed as bleeding other than major bleeding that affected the clinical status of the patient albeit to a lesser extent [9].

The development of chronic pulmonary thromboembolism (CPTE), chronic thromboembolic pulmonary hypertension (CTEPH), and recurrent pulmonary thromboembolism (Re-Emboli) were recorded in the patients groups [2, 9, 10]. These data were collected from medical records. These patients, who discharged from the hospital, are followed up in the outpatient clinic by pulmonary physicians independently of the study.

Mortality was evaluated as one-month and one-year all-cause mortality after the diagnosis time of PE.

### Collection and recording of study data

Patient data from the EGEPET period were collected from electronic records, while pre-EGEPET data were obtained retrospectively from electronic or written files. All data were recorded in a standardized form.

Age, gender, smoking status, comorbidities, PE risks, Charlson Comorbidity Index, admission clinical and laboratory parameters, and sPESI scores of all patients were recorded after applying inclusion and exclusion criteria. Systemic arterial blood pressure (BP), pulse, and oxygenation status on the admission day (before treatment) and second days of treatment; complications, and mortality status were evaluated. Relevant clinical parameters were systematically followed from the beginning of the end of the study.

### Primary and secondary outcomes

The primary outcomes were one-month and one-year mortality. Secondary outcomes included changes in vital signs (heart rate, BP) and oxygenation (PaO₂/FiO₂ ratio) following thrombolysis, together with hemorrhagic complications, Re-emboli, CPTE, and CTEPH.

### Statistics

Statistical analysis was made with SPSS (Statistical Package for Social Sciences) 20.0 program. The distributions of the variables were evaluated with the “Kolmogorov–Smirnov and Shapiro–Wilk test.” While numerical data were presented as mean ± standard deviation or median (Iq25-Iq75) value, categorical variables were presented as “%”. Parameters that fit the normal distribution were compared by Student-t test and the parameters that did not fit the normal distribution were compared by Mann–Whitney U test, which is a nonparametric test. Chi-square or Fisher’s exact test was used to compare categorical variables. The comparison of the parameters evaluated on the admission and second days of the treatment was performed with the Wilcoxon test. Multivariate analysis for one-month mortality was performed by including age, sex, thrombolytic therapy, and the parameters found to be associated with one-month mortality in the univariate analysis. The value of “*p* < 0.05” was accepted as statistically significant.

## Results

### Patient population

In the thrombolytic group, 59 patients who were diagnosed with intermediate-high risk PE during the EGEPET period and received half-dose thrombolytic therapy were prospectively assessed.

**Fig. 1:**
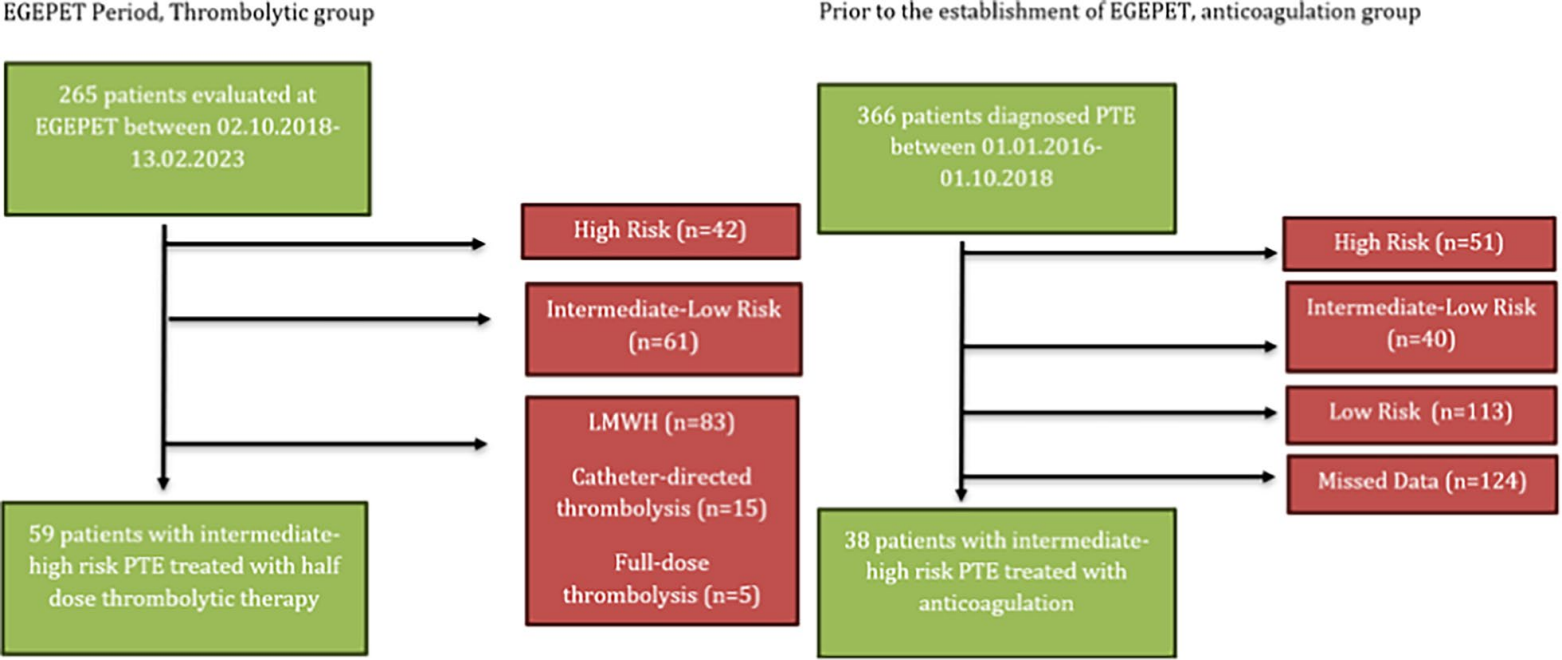
Identification of the patient population. Abbreviations: EGEPET: Ege Pulmonary Emboli Team; LMWH; Low Molecular Weight Heparin, PTE: Pulmonary Thromboembolism

For the anticoagulant group, medical records of 366 patients diagnosed with PE and hospitalized between January 2016 and October 2018 were retrospectively screened. Among them, 38 patients met the criteria for intermediate-high risk PE and were included in the study (Fig. 1).

### Patients’ demographic and clinical characteristics

PE was diagnosed by CTPA in the majority of patients (98%). Only one patient underwent V/P scintigraphy. Another patient, who was morbidly obese and could not undergo CT, was diagnosed with PE based on the presence of RV dysfunction on ECHO and deep vein thrombosis on Doppler ultrasonography.

The median age of the two groups was similar (66 and 68 years). However, the proportion of female patients was higher in the thrombolytic group compared to the anticoagulation group (45.8% vs. 68.4%, *p* = 0.037). All patients in the thrombolytic group had bilateral PE, while 35 patients (92.1%) in the anticoagulation group had bilateral PE (*p* = 0.59).

**Fig. 2:**
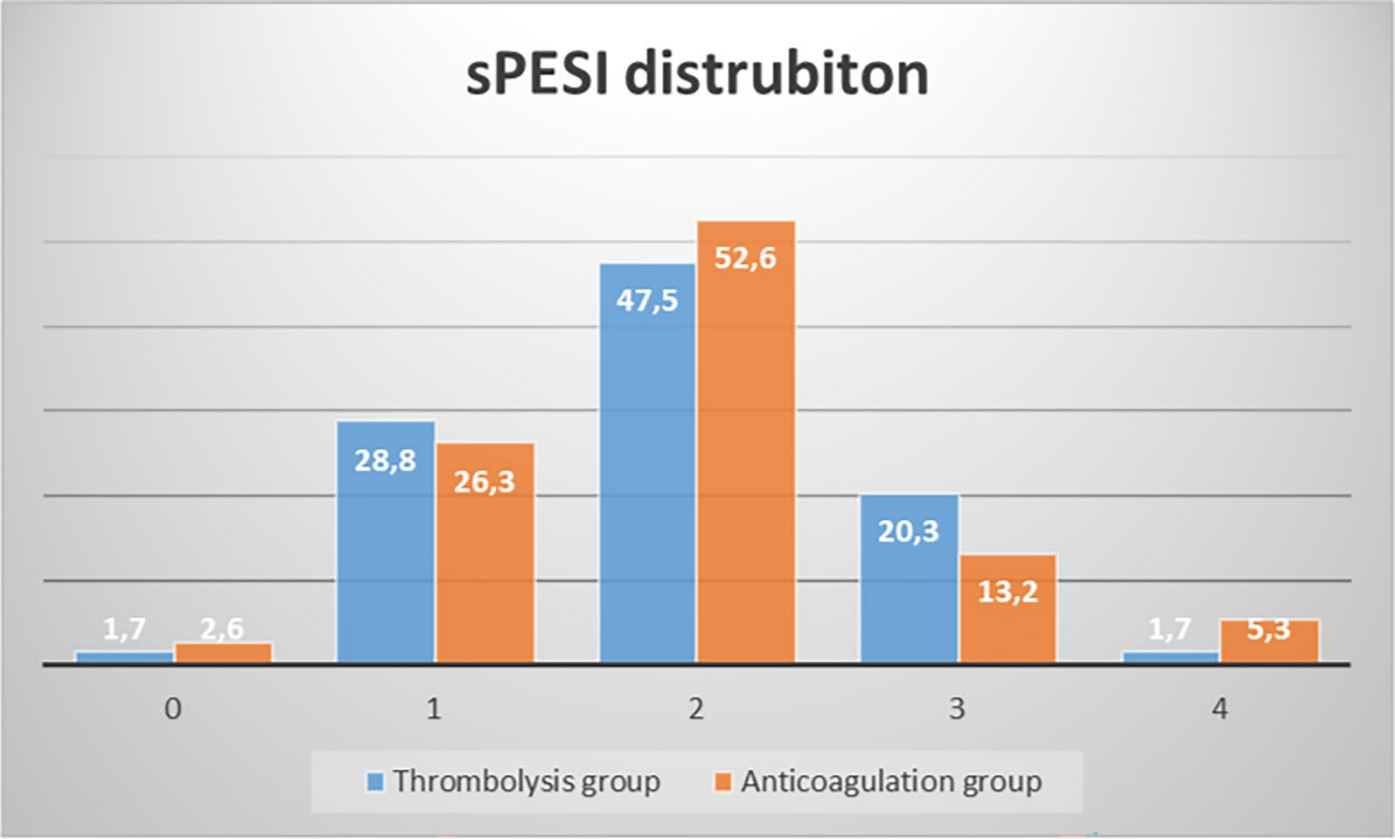
The distribution of sPESI scores of the cases in the thrombolytic and anticoagulation groups

There was no difference between the two groups regarding comorbidities, sPESI score, RIETE score, HAS-BLED score, and history of recurrent embolism. The median Charlson comorbidity Index value was 4 (2.0–5.75) in the anticoagulation group, whereas it was 3 (1.0–5.0) in the thrombolytic group (*p* = 0.050) (Table 1). Figure 2 presents the sPESI distribution of both groups.

**Table 1:** Demographic and clinical characteristics of the cases in the thrombolytic group and the anticoagulation group

Parameters	Thrombolytic Group (n = 59)	Anticoagulation Group (n = 38)	p value
Age, (year)*	66 (51.0–73.0)	68 (59.5–79.25)	0.054
Female, *n (%*)	27 (45.8)	26 (68.4)	**0.037**
Hypertension, *n (%*)	30 (50.8)	20 (54.1)	0.84
Diabetes Mellitus, *n (%*)	11 (18.6)	8 (21.6)	0.79
Malignancy, *n (%*)	9 (15.3)	7 (18.4)	0.78
Dementia, CVD, Alzheimer, Parkinson, *n (%*)	7 (11.9)	6 (15.8)	0.76
Coronary Artery Disease, *n (%*)	6 (10.2)	4 (10.8)	1.0
CHF, *n (%*)	1 (1.7)	2 (5.3)	0.56
Hypothyroidism, *n (%*)	3 (5.1)	2 (5.3)	1.0
Rheumatism Disease, *n (%*)	3 (5.1)	3 (8.1)	0.67
COPD, *n (%*)	3 (5.1)	2 (5.3)	1.0
IPF, *n (%*)	0	1 (2.6)	0.39
Renal Failure, *n (%*)	1 (3.4)	1 (2.7)	1.0
Charlson Comorbidity Index*	3.0 (1.0–5.0)	4 (2.0–5.75)	0.050
sPESI score*	2.0 (1.0–2.0)	2.0 (1.0–2.0)	0.95
RIETE score*	2.0 (1.0–2.5)	2.50 (1.0–3.275)	0.10
HAS-BLED*	2.0 (1.0–2.0)	2.0 (1.0–2.0)	0.49
D-dimer (µg/L)*	4500 (4321–4572)	4281 (4249–4336)	** < 0.001**
Troponin (ng/L)*	55.0 (36.0–133.0)	62.0 (32.0–99.0)	0.36
Creatinine (mg/dL)*	1.0 (0.8–1.21)	0.92 (0.77–1.22)	0.22
Hemoglobin (g/dL), (mean ± SD)	13.82 ± 2.12	12.35 ± 2.12	**0.001**
Platelet count (/µL)*	218000 (180000–275000)	236000 (194750–306750)	0.21

*CHF* congestive heart failure, *COPD* chronic obstructive pulmonary disease, *CTPA* computed thorax pulmonary angiography, *CVD* cerebrovascular disease, *IPF* idiopathic pulmonary fibrosis, *SD* standard deviation, *sPESI* simplified pulmonary embolism severity index

^*^median (Iq25-Iq75)

Comparison of PE risk factors between the two groups revealed that immobility was more common in the anticoagulation group than in the thrombolytic group (44.7% vs. 22%, *p* = 0.013). No contraindications for thrombolysis were identified in patients with a history of surgery or trauma.

### Patients’ vital signs

The thrombolytic and anticoagulation groups were similar in terms of systolic BP, mean arterial pressure, and PaO₂/FiO₂ ratio measured at admission before treatment. However, the pulse rate at admission was higher in the thrombolytic group (Table 2).

**Table 2:** Vital signs of the thrombolytic and anticoagulation group on the admission day, before treatment

Parameters	Thrombolytic Group (n = 59)	Anticoagulation Group (n = 38)	*p* value
Admission day systolic BP, *(mmHg)*	121 (105–130)	122 (112–140)	0.14
Admission day mean BP. *(mmHg)*	88 (80–100)	89 (85–100)	0.45
Admission day pulse /min	116 (105–127)	107 (86–121)	**0.032**
Admission day PaO_2_/FiO_2_	330 (270–380)	293 (255–389)	0.45

All data are provided as median (Iq25-Iq75)

*BP* blood pressure, *PaO*_*2*_*/FiO*_*2*_* ratio* arterial partial oxygen pressure/fractionated oxygen ratio

Table 3 presents the changes in vital signs observed in each group after treatment. In the thrombolytic group, significant improvements were observed in both oxygenation (*p* < 0.001) and pulse rate (*p* < 0.001) on the second day. In contrast, the anticoagulation group showed a significant decrease only in pulse rate (*p* < 0.001). No significant changes were observed in BP measurements in either group (Table 3).

**Table 3:** Evaluation of the differences in the vital signs of patients in thrombolytic and anticoagulation groups on the admission day and the second day

	Thrombolytic Group (n = 59)			Anticoagulation Group (n = 38)		
Parameters	Admission Day	Second Day	p value	Admission Day	Second Day	*p* value
Systolic BP, *(mmHg)*	121 (105–130)	123 (114–132)	0.15	122 (112–140)	128 (111–141)	0.64
Mean BP. *(mmHg)*	88 (80–100)	87 (82–95)	0.82	89 (85–100)	92 (80–101)	0.63
Pulse /min	116 (105–127)	91 (80–104)	** < 0.001**	107 (86–121)	94 (80–101)	** < 0.001**
PaO_2_/FiO_2_	330 (270–380)	417 (351–447)	** < 0.001**	293 (255–389)	351 (263–384)	0.27

All data are provided as median (Iq25-Iq75)

*BP* blood pressure, *PaO*_*2*_*/FiO*_*2*_* ratio* arterial partial oxygen pressure/fractionated oxygen ratio

### Comparison of complications and mortality

No statistically significant differences were observed between the two groups in terms of treatment-related complications. No major bleeding events occurred. The duration of hospitalization was similar between the groups; however, the length of stay in the intensive care unit was significantly shorter in the anticoagulation group compared to the thrombolytic group (*p* < 0.001) (Table 4).

**Table 4:** Mortality rates and long-term complications in the thrombolytic group and anticoagulation group

Parameters, *n (%)*	Thrombolytic Group (n = 59)	Anticoagulation Group (n = 38)	*p* value
One-*month* mortality	4/59 (6.7)	6/38 (15.8)	0.18
One-*year* mortality *	7/51 (13.7)	10/38 (26.3)	0.17
Chronic Pulmonary Thromboembolism **	4/43 (9.3)	1/23 (4.3)	0.65
Chronic Thromboembolic Pulmonary Hypertension **	2/43 (4.3)	1/23 (4.3)	1.0
Re-embolism ***	1/46 (2.2)	3/32(9.4)	0.30
Complication present	5 (8.5)	2 (5.3)	0.70
*Complication types*			
Hematoma	1 (1.7)	–	
Hematuria	1 (1.7)	1 (2.6)	
Unexplained hemoglobin drop	1 (1.7)	–	
Hemoptysis	2 (3.4)	–	
HIT	–	1 (2.6)	
In-hospital mortality	4 (6.7)	5 (13.1)	0.73
Duration of intensive care unit *(day)* median (Iq25-Iq75)	3.0 (2.0–4.0)	1.5 (0–2.25)	** < 0.001**
Duration of hospital stay *(day)* median (Iq25-Iq75)	9.0 (6.0–11.0)	8.0 (6.0–12.0)	0.69

*One-year mortality was evaluated in 51 patients in the thrombolytic group whose follow-up period was completed, and in all patients in the anticoagulation group

**Patients with missing data were not included in this analysis, only 66 patients were assessed

***Re-embolism: There were 78 patients followed for recurrent embolism

Abbreviations: HIT: Heparin Induced Thrombocytopenia

The choice of anticoagulant for maintenance therapy and the duration of treatment were determined by the attending physician based on the underlying causes. No significant differences were observed between the groups regarding the development of CPTE and CTEPH. A total of 43 patients in the thrombolytic group and 26 in the anticoagulation group were monitored for these outcomes. One-month mortality was 6.8% in the thrombolytic group and 15.8% in the anticoagulation group, while one-year mortality was 13.7% and 26.3%, respectively (*p* = 0.17). Although mortality rates were lower in the thrombolytic group, the difference was not statistically significant (Table 4).

In the multivariate analysis, age > 67 (OR: 8.82, %95 CI 1.54 – 50.53 *p* = 0.014), second-day pulse rate > 94/min (OR 7.61, 95% CI 1.33–43.49, *p* = 0.022) were associated with a significantly higher risk of one-month mortality. Thrombolytic therapy, male sex, second-day PaO₂/FiO₂ > 360 were not significantly associated with mortality (Table 5).

**Table 5:** Multivariate Analysis of Factors Associated with One-Month Mortality

Parameters	*p* value	Odds ratio	%95 CI OR
Thrombolytic Therapy	0.79	1.26	0.23—7.06
Gender (male)	0.78	1.25	0.26 – 6.17
Age > 67	0.014	8.82	1.54 – 50.53
Second day pulse > 94/min	0.022	7.61	1.33 – 43.49
Second day PaO2/FiO2 > 360	0.137	4.34	0.62 – 30.12

*CI* confidence interval, *OR* odds ratio

#### Post-hoc power analysis

Post-hoc power analysis based on the observed difference in one-month mortality (6.7% vs. 15.8%) yielded a statistical power of 29.3%. This indicates that the study was underpowered to detect a significant difference, despite the numerically lower mortality in the thrombolytic group.

## Discussion

This study compared half-dose alteplase and low molecular weight heparin (LMWH) as initial treatment options in patients with intermediate-high risk PE. Although one-month and one-year mortality rates were lower in the thrombolytic group, the differences were not statistically significant. To our knowledge, this is the first real-life study comparing these two agents in this specific risk group. No significant differences were observed between the groups regarding major bleeding, minor bleeding, or long-term complications. The multivariate analysis demonstrated that advanced age and increased heart rate on the second day were independently associated with one-month mortality, while thrombolytic therapy, gender, and second-day PaO_2_/FiO_2_ ratio were not found to be as risk factor.

Intermediate-high risk PE is associated with greater mortality than intermediate-low-risk PE. While the ESC/ERS guidelines recommend anticoagulation and close monitoring for these patients, fibrinolytic therapy is suggested only in cases of hemodynamic deterioration. Similarly, the American Society of Hematology advises against routine thrombolytic use in hemodynamically stable patients with right heart strain. This cautious approach is primarily due to insufficient supporting evidence [2, 11]. On the other hand, the use of thrombolytics for intermediate-high risk PE patients is often discussed by “Pulmonary Embolism Response Teams” [12, 13]. In our clinical practice, patients in this group are treated with half-dose thrombolytic therapy after the establishment of EGEPET unless contraindicated.

Mirambeaux et al. followed 1015 hemodynamically stable patients prospectively. According to their results, intermediate-high risk PE was found in 97 (9.6%) of the patients based on the ESC/ERS PE guideline, and The one-month all-cause mortality was 24% in this group [14]. The mortality rate is important and too high to be ignored. In our presented study, the mortality rate was 6,7% in the thrombolytic group and 15,8% in the anticoagulation group. However, the contribution of the reperfusion therapy to low mortality rate in our series is not sufficient to suggest because of the limited number of patients.

Arterial hypotension, tachycardia, low partial oxygen pressure, and low oxygen saturation parameters are seen as risk factors for short-term (30-day) mortality, and in addition to hemodynamic instability [15, 16]. Declined BP and PaO_2_/FiO_2_ ratio, and increased pulse value are accepted as poor prognosis signs during follow-up. In our study, vital signs of patients were similar in both groups on admission; but after half-dose thrombolytic treatment, dramatic improvement was seen in pulse rate and in PaO_2_/FiO_2_ value. These results point to clinically rapid improvement in the thrombolytic group. The observed improvements in vital signs—specifically, the significant enhancements in PaO₂/FiO₂ ratios and reductions in pulse rates—suggest a rapid physiological response to half-dose thrombolytic therapy. These findings may indicate early reversal of right ventricular dysfunction and improved pulmonary perfusion, which are critical in the management of intermediate-high risk PE patients. Although the improvement in PaO₂/FiO₂ ratio suggests enhanced oxygenation following thrombolysis, this marker should be interpreted with caution, as it may not directly point clinical endpoints such as mortality or recurrence. In recent years, that patients in this PE classification are closely followed up because of disease severity and thrombolytic therapy. Therefore, the longer ICU day observed in the thrombolytic group may reflect differences in treatment approach and raised awareness of clinical severity.

Both thrombolytic therapy and anticoagulation therapy have well-recognized complications. Meta-analysis made by Zuo et al. has shown that thrombolysis increases major bleeding by 2.84 times and minor bleeding by 2.97 times compared to the anticoagulation [17]. Although thrombolytic therapy increases the risk of bleeding, it corrects signs of right ventricle failure, rapidly and effectively [4]. In this situation, the decision to use thrombolytic therapy becomes an important and difficult clinical problem. And that should be remembered the characteristics of the patient group in studies is important for evaluating results.

Riera-Mestre et al. analyzed the efficacy and side-effects of the thrombolytic therapy in their meta-analysis composed of eleven studies. Different thrombolytic agents were assessed in here research results showed that thrombolysis did not reduce mortality risk, but increased bleeding risk. With these results, the authors do not recommend the use of thrombolytics in intermediate-risk PE [18]. In this meta-analysis, the patients were not classified as intermediate-high and intermediate-low risk according to new guidelines. However, there are also other studies reporting that thrombolytic therapy does not increase the risk of bleeding compared to anticoagulation therapy. [19]. In the presented study, no major bleeding was observed, and no difference was found between the anticoagulant (5.3%) and thrombolytic (8.5%) groups in terms of minor bleeding.

Meyer et al. compared tenecteplase (30–50 mg) with unfractionated heparin in PE patients with right ventricular failure and elevated troponin. The primary endpoint was hemodynamic decompensation within 7 days, which occurred less frequently in the thrombolytic group (2.6% vs. 5.6%, statistically significant). However, this difference was not significant in patients younger than 75 years. Major bleeding events were more common in the thrombolytic group. The 30-day mortality rates were 2.4% and 3.2% in the thrombolytic and anticoagulation groups, respectively, without a significant difference. Although case definitions were similar, patients with hemodynamic decompensation during follow-up and those receiving full-dose thrombolysis were excluded from our study. Additionally, different thrombolytic agents were used [4].

The MOPETT study compared the efficacy of thrombolytic therapy with low-dose tissue plasminogen activator and anticoagulant in patients with intermediate-risk. Heparin infusion or enoxaparin was administered for anticoagulation therapy. In MOPETT trial, intermediate risk PE was defined as clinical findings of PE and the presence of severe radiological PE. The hospital mortality rate was 1.6% in the thrombolytic group and 5% in the anticoagulation group. While no recurrent embolism occurred in the thrombolytic arm, 5% of the anticoagulation arm had recurrent embolism (*p* = 0.08). No bleeding was observed in both groups. The hospital mortality rate in our study and the frequency of recurrent emboli were higher compared to MOPETT study. Recurrent embolism rate in both studies was lower in the thrombolytic arm than the anticoagulation arm. In the MOPETT study, right ventricle failure and troponin positivity were not assessed, the patients were classed into only radiological findings rather than clinical severity. [20]. Moreover, our patient group was older than MOPETT trial group and was evaluated with Charlson comorbidity index. In MOPETT, while there was no difference between the groups in terms of the presence of one-to-one comorbidity, the burden of all comorbidities did not seem to be evaluated.

In Türkiye, Yılmaz et al. compared the efficacy of LMWH and 50 mg alteplase in patients with PE who were hemodynamically stable but had right ventricle failure findings in CTPA or ECHO. There were 38 patients in each group. One death (3%) occurred in the thrombolytic group within 30 days, while death was observed in 4 (10%) patients in the anticoagulation group (*p* = 0.36). Additionally, hemodynamic decompensation/death was observed in 1 patient in the thrombolytic group and in 10 patients in the anticoagulation group (*p* = 0.009). In other words, one-month mortality was not affected by half-dose alteplase, but the development of hemodynamic decompensation could be prevented. Although similar in design to our study, biomarkers were not used in this study as indicators of cardiac damage [6].

Guru PK et al. investigated ultra-low-dose systemic tPA (25 mg) in 4 patients with high risk submassive PE and found that even significantly reduced doses could yield clinical benefit without markedly increasing the bleeding risk [21]. Their focus was primarily ultra-low dose tPA application. Krishnan AM et al. compared catheter-directed thrombolysis with systemic thrombolysis and anticoagulation alone in patients with acute PE and cor pulmonale. They noted better hemodynamic recovery and RV function improvement in the thrombolysis group [22]. These findings indicate that alternative treatments strategies and different dosing may be considered for this patient population in the future.

Findings of RV failure and large thrombus in the pulmonary arteries increase the risk of CTEPH [23]. The results of the study following 219 patients with intermediate risk PE presented that 13.2% of patients develop right ventricular dysfunction on ECHO after CTEPH status or post-PE impairment [24]. Although thrombolytic therapy is thought to be protective against CPTE or CTEPH, more data is needed on this subject. When the MOPETT study was analyzed in terms of the development of pulmonary hypertension (systolic pulmonary artery pressure above 40 mmHg on ECHO), the rate of development of pulmonary hypertension during follow-up was 57% in the group receiving anticoagulation and 16% in the group receiving thrombolytics (*p* < 0.001) [20]. In the present study, the rates of CPTE or CTEPH were the same between the two groups, but it should be remembered that the follow-up period was longer in patients receiving anticoagulants.

Limitations:

This single-center study, investigating half-dose alteplase efficacy, has limitations. First, its single-center, real-life observational design limits generalizability and may make it prone to observer bias. Second, the thrombolytic cases were prospectively followed; but anticoagulant group data were collected retrospectively as a historical control group. The observed improvements in the thrombolytic group should be interpreted with caution, as the use of a historical control group may have introduced selection and documentation bias, potentially reflecting advances in multidisciplinary evaluation (EGEPET) and supportive care over time rather than the intervention itself. It should also be noted that the observed improvements may partially reflect the impact of systematic, team-based management introduced with the establishment of EGEPET. Third, the targeted sample size was not reached, with only 97 patients over seven years due to the rarity of this group. The study may be underpowered to detect statistically significant differences in outcomes such as mortality, and several p-values approaching significance raise the possibility of a type II error. Fourth, in the multivariable analysis, the confidence intervals of the parameters found to be statistically significant were detected as widely. They should be evaluated carefully. Fifth, it was not a randomized controlled trial, but baseline demographics and clinical findings were comparable. Sixth, patients requiring dose escalation or with deteriorating hemodynamics were excluded. Lastly, follow-up during the COVID-19 pandemic (2020–2021) was challenging. Despite these limitations, this study reflects real-life clinical practice.

## Conclusion

The use of anticoagulants versus thrombolytics in intermediate-high risk acute PE remains controversial. Guidelines suggest initial anticoagulation, reserving thrombolytics for hemodynamic decompensation, but evidence is limited. Recent studies highlight experiences with Pulmonary Embolism Response Teams and thrombolytic therapy in stable patients with RV failure. Our study, the first to compare 50 mg alteplase and LMWH in this group, found a statistically non-significant but numerically favorable improvement in PaO₂/FiO₂ ratio with thrombolytics. No major bleeding occurred in either group. Although one-month and one-year mortality rates were lower in the thrombolytic group, this difference did not reach statistical significance. Studies with large number patients are needed to clarify the potential benefit of half-dose thrombolysis in this special group.

## Data Availability

No datasets were generated or analysed during the current study.

## Abbreviations

BP: Blood pressure
CHF: Congestive heart failure
COPD: Chronic obstructive pulmonary disease
CPTE: Chronic pulmonary thromboembolism
CTEPH: Chronic thromboembolic pulmonary hypertension
CTPA: Computed thorax pulmonary angiography
CVD: Cerebrovascular disease
ECHO: Echocardiography
EGEPET: Ege pulmonary embolism team
ESC/ERS: European society of cardiology/European respiratory society
HIT: Heparin-associated thrombocytopenia
IPF: Idiopathic pulmonary fibrosis
LMWH: Low molecular weight heparin
PaO₂/FiO₂ ratio: Arterial partial oxygen pressure/fractionated oxygen ratio
PE: Pulmonary embolism
Re-Emboli: Recurrent pulmonary thromboembolism
RV: Right ventricle
sPESI: Simplified pulmonary embolism severity index
SD: Standard Deviation
V/P: Scintigraphy: Ventilation/Perfusion Scintigraphy
